# Large Brunner’s gland adenoma of the duodenum for almost 10 years

**DOI:** 10.1515/biol-2020-0029

**Published:** 2020-04-26

**Authors:** Bo Yang, Ke Li, Runlan Luo, Zuming Xiong, Lianwei Wang, Jinming Xu, Dengyang Fang

**Affiliations:** Department of General Surgery, Fuling Central Hospital of Chongqing City, Chongqing, 408000, China; Department of Ultrasound, Fuling Central Hospital of Chongqing City, Chongqing, China

**Keywords:** duodenum, Brunner’s glands, adenoma, benign tumor

## Abstract

**Background:**

Brunner’s gland adenoma is a rare benign tumor arising from Brunner’s glands. It is mostly small in size, and patients with this tumor are asymptomatic.

**Case presentation:**

We report the case of a 63-year-old woman with upper gastrointestinal obstruction for almost 10 years, who was pathologically diagnosed with large Brunner’s gland adenoma of the duodenum. Postoperatively, no sign of recurrence has been noted until now.

**Conclusion:**

This study may help clinicians to understand and provide a more accurate diagnosis of Brunner’s gland adenoma.

## 1 Introduction

Johan Conrad Brunner, a Swedish anatomist, first described Brunner’s glands in 1688. However, Brunner’s gland adenoma, also called polypoid hamartoma or Brunneroma, was first described by Curveilheir in 1835. It is a rare benign tumor arising from Brunner’s glands that may be transformed into a malignant tumor [1,2,3] and is mostly small in size. Patients with this tumor are asymptomatic. Occasionally, it may be large in size, which may cause hemorrhage or obstruction. Herein, we report the case of a patient who had large Brunner’s gland adenoma of the duodenum with upper gastrointestinal obstruction for almost 10 years and review the literature extensively.

## 2 Case report

**Figure 1:**
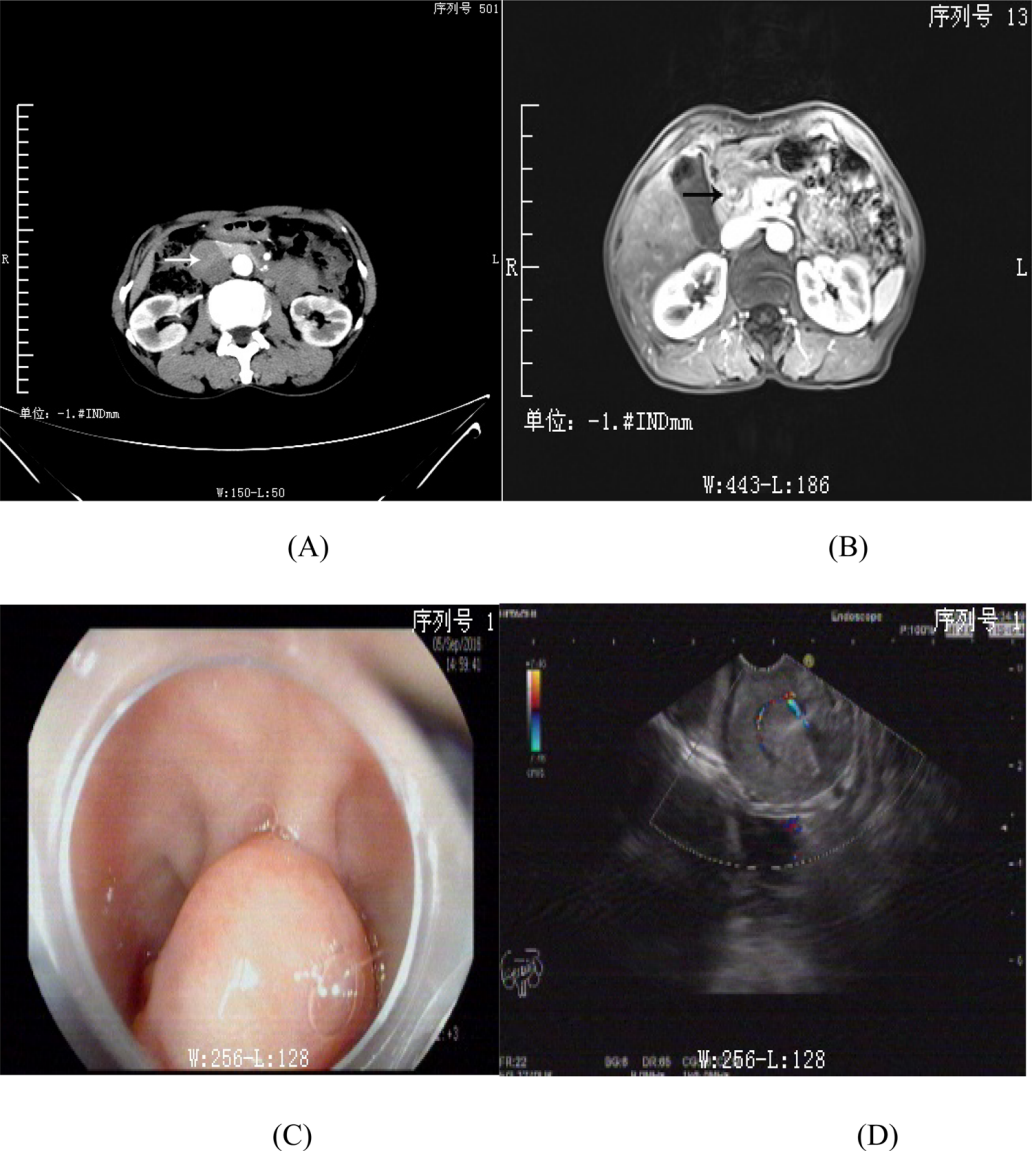
The abdomen CT (A), abdomen MRI (B), and endoscopic ultrasonography (C and D) of Brunner’s gland adenoma in this case. (A) Implied a uniform soft tissue mass shadow in the descending duodenum. (B) Revealed a significant thickening of the wall of the duodenal bulb and descending duodenum. (C and D) Demonstrated that there was a protrusion lesion with a clear boundary, smooth surface, and color signals in bulb duodenum.

A 63-year-old woman came to our hospital complaining of recurrent upper abdominal fullness discomfort for almost 10 years. She experienced exacerbation of intermittent nausea, vomiting with chyme (5 mL), heartburn, acid regurgitation, and eructation for 6 months. No history of hypertension, diabetes mellitus, or coronary heart disease was noted. Upon physical examination, a 5 × 4 cm mass with a hard texture and poor mobility was observed in the upper abdomen. Routine blood and tumor marker test results were within normal range. Abdomen computed tomography (CT, Figure 1A) showed a 32 mm × 22 mm soft tissue mass shadow with homogeneous density in the descending duodenum, which was protruding into the duodenal lumen. Moreover, thickening of the adjacent intestinal wall was noted. Upper abdomen magnetic resonance imaging (MRI, Figure 1B) revealed a significant thickening of the wall of the duodenal bulb and descending duodenum. The wall thickness was 1.1 cm. The signals were slightly low in *T*_1_-weighted images and slightly high in *T*_2_-weighted images. Endoscopic ultrasonography (Figure 1C and D) demonstrated that there was a protrusion in the duodenal bulb of about 26 × 18 mm in size with a clear boundary, smooth surface, and irregular shape and the base being about 17 mm, color signals abound.

**Figure 2:**
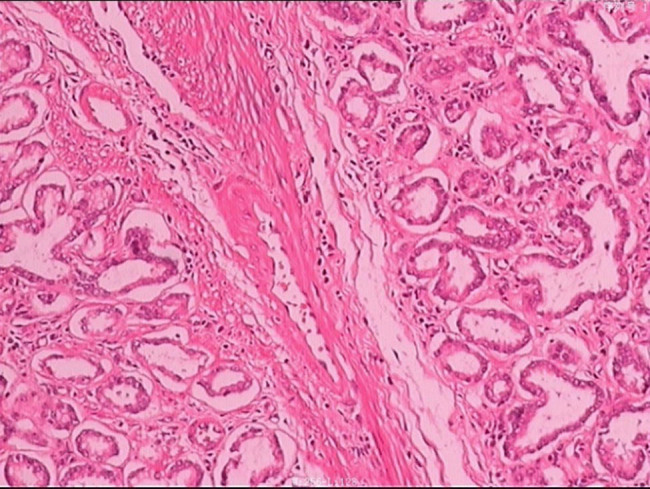
The pathology of this case. It revealed multiple Brunner’s glands with tubes, fibers, and smooth muscle diffuse distribution.


Neoplasm resection of duodenum was performed, and we found a mass measuring about 25 × 30 × 10 mm located in the descending duodenum. It was soft, brittle, and mobile with a clear boundary. The pathological result (Figure 2) of the mass revealed multiple Brunner’s glands with tubes, fibers, and smooth muscle diffuse distribution. No dysplasia was noted on the epithelium. It was diagnosed as Brunner’s gland adenoma of the duodenum. The patient was discharged from the hospital a week after recovery. To date, no relapse has occurred.

**Informed consent:** Informed consent has been obtained from the patient included in this study.

## 3 Discussion

Brunner’s gland adenoma is primarily located in the duodenum, especially the proximal duodenum, and is possibly caused by hyperplasia of the secretory tubes and stroma in the proximal duodenum [4,5]. Brunner’s gland could be classified into three types based on its size [6]: type I (diffuse nodular hyperplasia), which is confined to the mucosa with multiple sessile projections occupying most of the duodenum; type II (circumscript nodular hyperplasia), which is found in the bulb duodenum and is usually smaller than 1 cm; and type III (Brunner’s gland adenoma), which is usually stemmed and sized 1–2 cm, generally without clinical manifestations [7]. The etiology and pathophysiology of Brunner’s gland adenoma are unknown. The adenoma may be associated with increased acid secretion [8] or *Helicobacter pylori* infection [9]. It is reported that patients with chronic gastric erosion and duodenal ulcer are more prone to Brunner’s gland adenoma [10]. In our case, the patient suffered from chronic superficial gastritis. This is possibly a predisposing factor for Brunner’s gland adenoma, which usually occurs in individuals aged 50–60 years, without gender difference. Large Brunner’s gland adenomas of several centimeters in size are extremely rare [11] and may cause upper gastrointestinal hemorrhage and obstruction, vomiting, stomachache, diarrhea [12], anemia [13], acute pancreatitis, and obstructive jaundice [13,14]. In this report, we present the clinical findings of a patient who had a large Brunner’s gland adenoma in the upper abdomen for almost 10 years, which presented as upper gastrointestinal obstruction.

The accurate diagnosis of Brunner’s gland adenoma can be made through histopathological examination; however, such examination is difficult to perform preoperatively. Gastrointestinal endoscopy, CT, and other radiologic imaging methods are useful for identifying the cause of the clinical manifestations. Nonetheless, Brunner’s gland adenoma is easily confused with pancreaticoduodenal tumors because it is nonspecific, which may lead to difficulties in diagnosing and changes in the treatment strategy [15]. At present, surgical excision is the most effective treatment for Brunner’s gland adenoma. However, whether asymptomatic Brunner’s gland adenoma detected coincidentally should be excised or not is still unclear at present. Some studies demonstrate that treatment is not required, while others report that endoscopic or surgical resection is important in preventing the complications due to Brunner’s gland adenoma [16]. In our opinion, it is necessary to perform surgical or endoscopic resection because the tumor may cause serious complications, including acute hemorrhage and even shock in some cases [11]. Brunner’s gland adenoma is a benign tumor with a good prognosis. Some literature reviews demonstrate that a few of these tumors could be malignant [3], and therefore, warrant attention. In our study, a large Brunner’s gland adenoma of the duodenum with upper gastrointestinal obstruction for almost 10 years was noted. Postoperatively, no sign of recurrence has been noted.

## 4 Conclusion

Brunner’s gland adenoma is a rare benign tumor of the duodenum. It is also an insidious cause of upper gastrointestinal obstruction because some patients may present with upper gastrointestinal bleeding, and a few of these tumors could be malignant, and therefore, warrant attention. Endoscopic resection is the first treatment choice when the tumor is small or has a stem. Surgery is reserved for cases where endoscopic resection has failed or when the tumor is large.
